# A rare brainstem anaplastic astrocytoma

**DOI:** 10.1515/tnsci-2022-0233

**Published:** 2022-09-06

**Authors:** Rongjiao You, Xiangfa Liu, Songfa Chen, Lixi Tan, Aiqun Liu, Mingfan Hong, Zhongxing Peng

**Affiliations:** Department of Neurology, School of Clinical Medicine, The First Affiliated Hospital of Guangdong Pharmaceutical University, 19 Nonglinxia Road, Guangzhou, 510080, China

**Keywords:** anaplastic astrocytoma, brainstem, misdiagnosis

## Abstract

**Background:**

Anaplastic astrocytoma (AA) is rarely observed in the brainstem and the clinical symptoms and imaging manifestations vary, which present a great challenge to accurate clinical diagnosis.

**Case description:**

A 56-year-old woman, with a month-long history of nausea and vomiting, was first diagnosed with acute cerebral infarction and demyelinating disease. The patient showed negative results on enhanced magnetic resonance and ^18^F-fluorodeoxyglucose positron emission tomography-computed tomography, and the clinical symptoms were not typical, leading to early misdiagnosis.

**Conclusion:**

Finally, the patient was diagnosed with AA by pathological biopsy.

## 1 Introduction

Anaplastic astrocytoma (AA) accounts for only 6.7% of gliomas [1], which occurs mostly in the cerebrum followed by cerebellum, while AA in the brainstem is only 3.6% [1]. They can be difficult to diagnose, and are challenging to treat. Clinical studies of this diagnosis are few and generally small [2]. The incidence of AA in the Caucasian community is higher than that of the Asian and Black communities [3], besides, it is approximately 1.8 times higher in men than women [4]. AA has a grade III classification according to the World Health Organization (WHO) tumor classification [5], indicating that AA has a high degree of malignancy and a poor prognosis. Therefore, early recognition of brainstem malignancy and timely treatment are essential. Brainstem AA is extremely rare in Asian women. Due to its atypical early symptoms, it is difficult for many doctors to recognize and is often misdiagnosed as stroke or brainstem demyelination disease, resulting in delayed treatment.

Here we report a case of an adult female with ^18^F-fluorodeoxyglucose (^18^F-FDG) positron emission tomography-computed tomography (PET-CT)-negative brainstem AA, which was misdiagnosed.

## 2 Case presentation

A 56-year-old woman presented with a month-long history of nausea and vomit with increased shallowness of the left nasolabial sulcus for 2 days. The patient visited a different local hospital a month ago where they could not find a cause for her symptoms. The patient’s vomiting and headache became worse during the treatment and the patient was transferred to our hospital for further treatment. The patient did not report anorexia and her body weight did not change significantly.

The patient had a history of uterine leiomyoma and did not have a recent history of cold, fever, diarrhea, or clinically significant family history. The patient did not have any history of brainstem tumor. Her family history was unremarkable.

Physical examination on admission revealed that the patient had central facial paralysis, obvious impairment of bilateral eye movement function, and gross horizontal nystagmus.

Cerebrospinal fluid (CSF) collected via lumbar puncture was colorless and transparent with a pressure of 140 mm H_2_O. The white blood cell count in the CSF was 3 × 10^6^/L, with 1.8 × 10^6^/L of lymphocytes, 1.1 × 10^6^/L of monocytes, and 0.1 × 10^6^/L of other white blood cells. The protein concentration was 0.35 g/L, and Pandy’s reaction was negative. The glucose and chloride ion levels were 4.66 and 120 mmol/L, respectively. The CSF was negative for aquaporin-4, myelin oligodendrocyte glycoprotein, and myelin basic protein antibodies.

**Figure 1:**
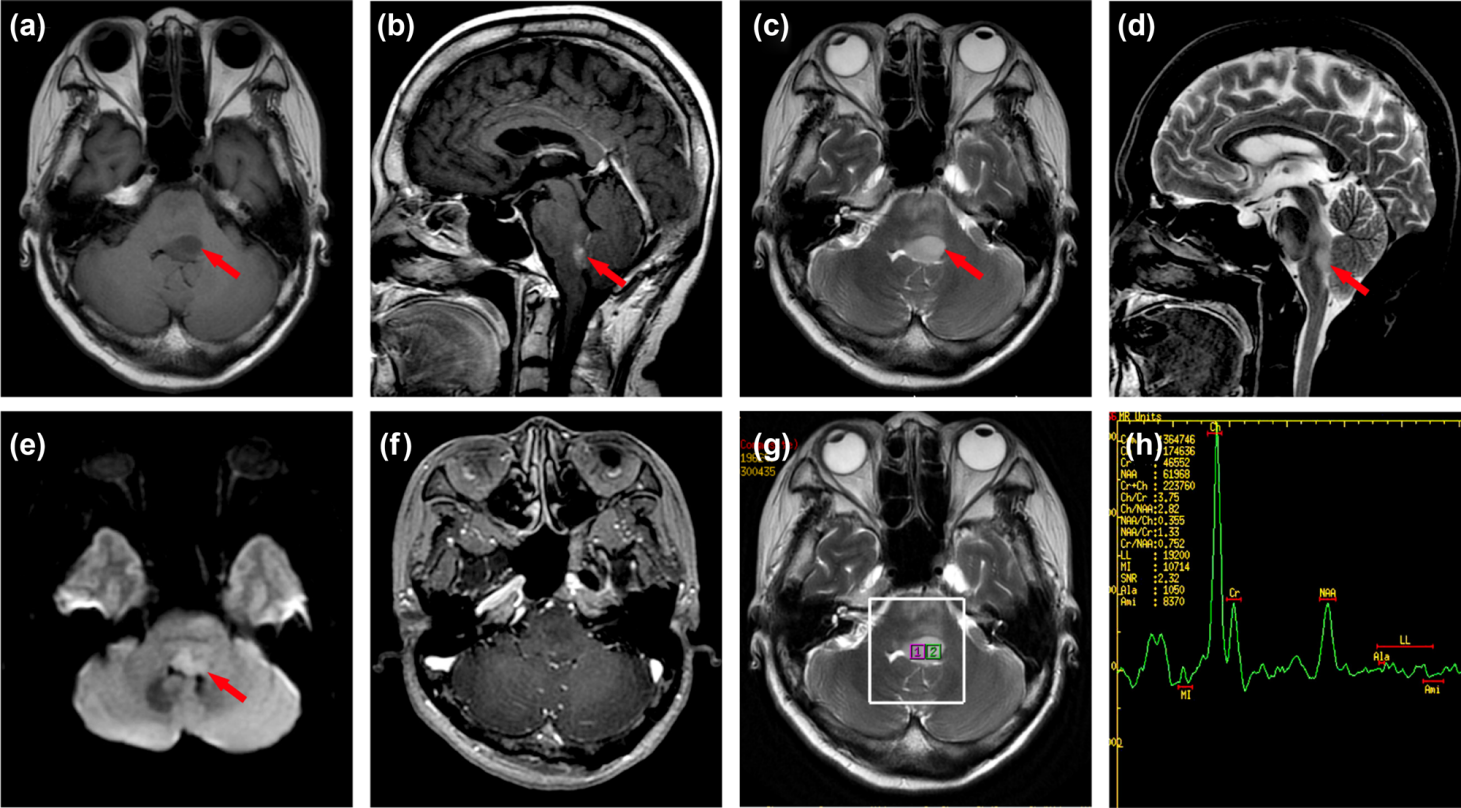
The patient’s first cranial MRI results show that the brainstem is enlarged and swollen. (a) T1WI revealed a mass of abnormal low signal intensity in the brainstem near the fourth ventricle (arrow). (b) T1-FLAIR, (c) T2WI, (d) T2-FLAIR, and (e) DWI showed that the abnormal mass in the brainstem had high signal intensity (arrows). (f) There was no obvious enhancement of the abnormal signal. (g and h) Cho/NAA = 2.83, Cho/Cr = 3.75, NAA/Cr = 1.33 of MRS point of interest 2.

**Figure 2:**
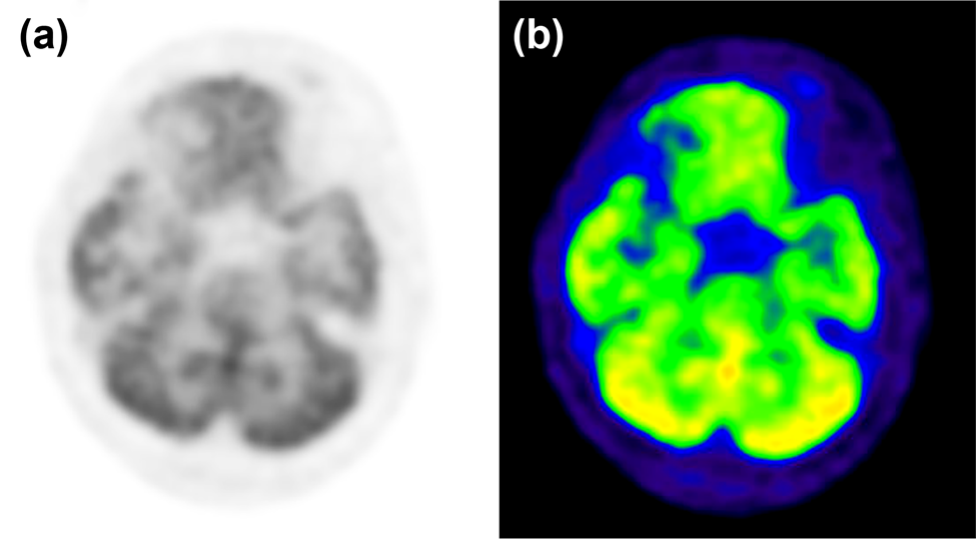
PET-CT (a) and ^18^F-FDG (b) did not reveal any obvious abnormalities.

**Figure 3:**
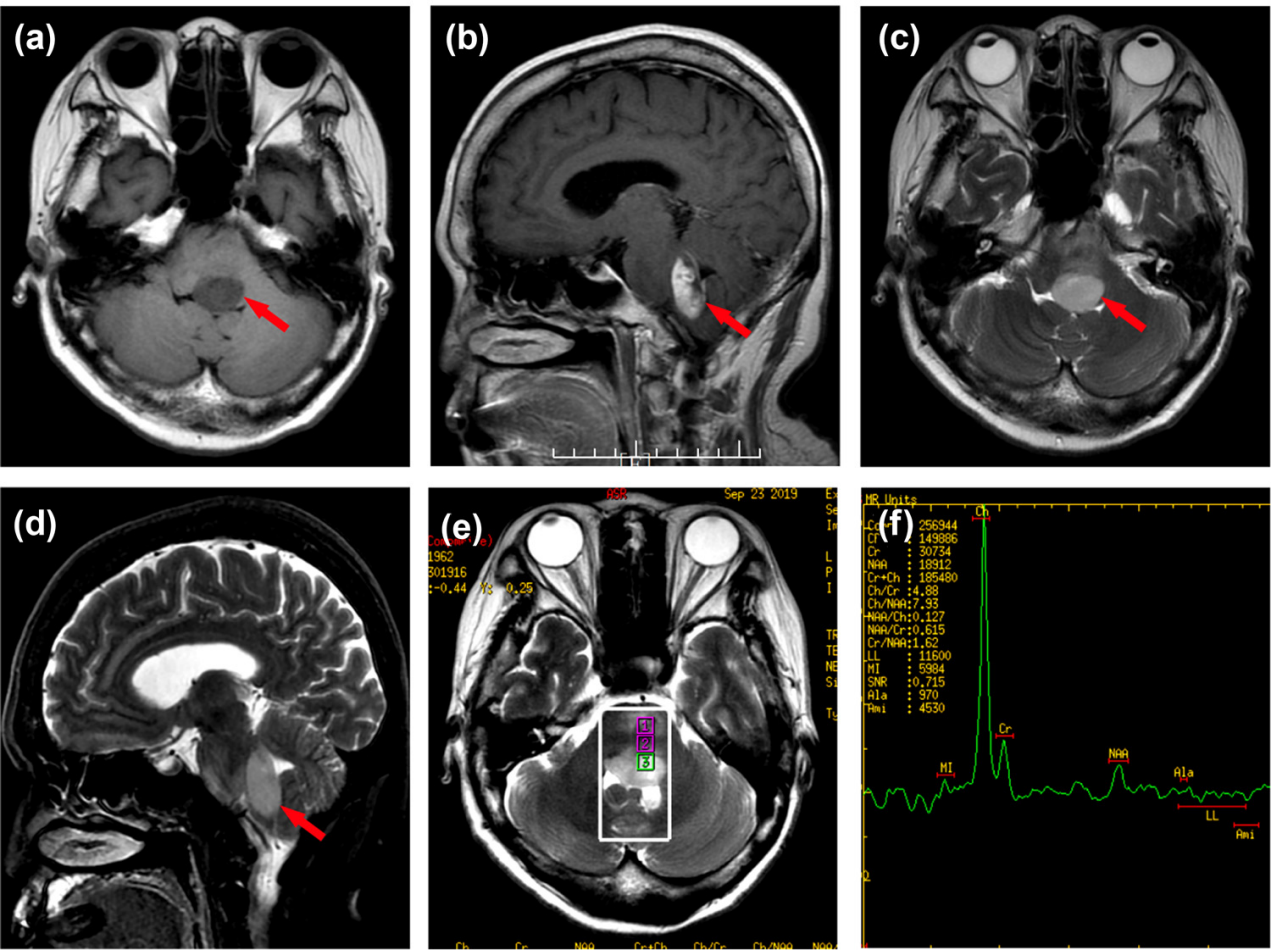
MRI performed after 4 weeks. (a) T1WI revealed that the further enlargement of abnormal low signal intensity (arrow). (b) T1-FLAIR, (c) T2WI, and (d) T2-FLAIR revealed that the further enlargement of abnormal high signal intensity in the brainstem and the compression on the surrounding brain tissue was more obvious (arrows). (e and f) Cho/NAA = 7.93, Cho/Cr = 4.88, NAA/Cr = 1.21 of MRS point of interest 3.

**Figure 4:**
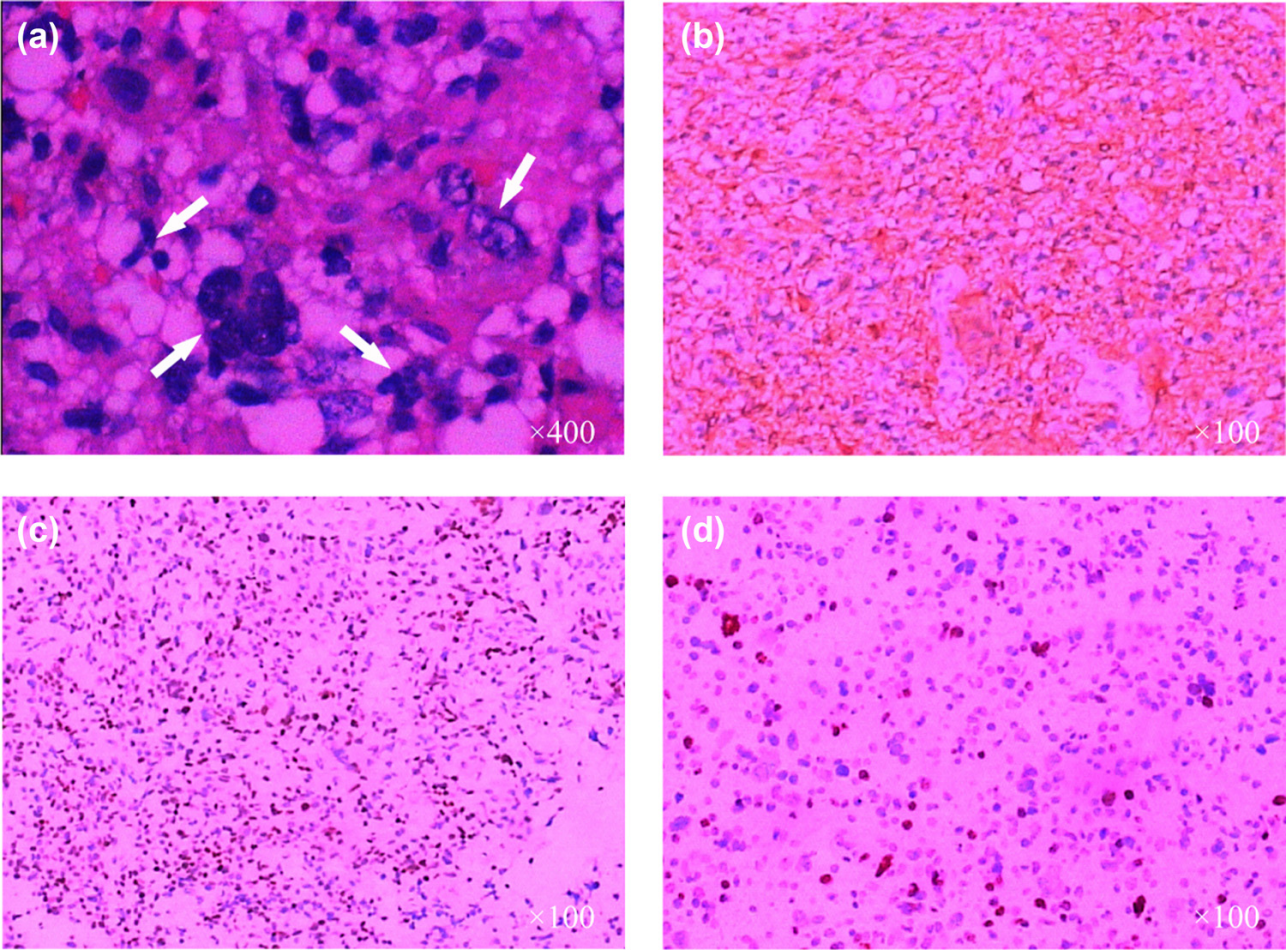
Figure showing the pathological picture of the lesion after the operation. (a) Hematoxylin and eosin staining of this lesion shows high local cell density, a rich nest cytoplasm, large cell body, multinucleated giant cells, and degenerative cells (arrows). (b and c): Immunohistochemical staining shows that the tumor cells are positive for GFAP (b) and p53 (c). (d) The Ki-67 labeling index is approximately 50%.

Magnetic resonance imaging (MRI) of the head revealed acute pontine infarction (lack of imaging data). Urgent head computed tomography (CT) imaging after admission revealed a thickened brainstem, although the nature of the lesion was unknown. The results of the cranial MRI (Figure 1) indicated that the patient had demyelinating lesions of the brainstem. Because of the space occupying effect of the lesion, the possibility of neoplastic lesions (lymphoma) could not be excluded. ^18^F-FDG PET-CT revealed no obvious signs of tumor (Figure 2). Four weeks later, a head MRI scan (Figure 3) revealed a high-grade astrocytoma which differed from a lymphoma. The space-occupying brainstem lesions were resected to make a definitive diagnosis and determine the appropriate treatment, and the frozen section obtained during the operation revealed a high-grade glioma. The final histopathology findings were consistent with an AA (WHO grade III) (Figure 4).

Follow-up: At 6 months follow-up, the patient was still alive, bedridden, and unable to take care of herself.


**Ethical approval:** The research related to human use has been complied with all the relevant national regulations, institutional policies and in accordance with the tenets of the Helsinki Declaration, and has been approved by the authors’ institutional review board or equivalent committee.
**Informed consent:** Informed consent has been obtained from all individuals included in this study.

## 3 Discussion

According to 2016 WHO classification guidelines for tumors of the central nervous system [5], AA is a grade III tumor that primarily occurs in cerebrum followed by the cerebellum. Hiep et al. [6] described a rare, pediatric, fourth-ventricular AA. Thus, AA in brainstem is relatively uncommon.

The clinical manifestations of patient with brainstem tumors vary with the lesion site and growth pattern. The focality and location of the lesion is distinguished from the clinical history, presentation, and associated imaging. In this case, the patient’s early symptoms were nausea and vomiting, rather than the usual triad of ataxia, long bundle syndrome, and multiple brain nerve damage [2]. The initial disease presentation demonstrated that the clinical symptoms may precede the appearance of imaging abnormalities due to the narrow space in the brainstem. Cases of brainstem blastoma have been reported in which patients have clinical symptoms, but prophase images are normal [7]. Brainstem lesions are often life-threatening, so early identification of these patients is crucial.

In this patient, T1WI showed low signal, T2WI FLAIR and diffusion-weighted imaging (DWI) revealed an enhanced signal, although enhanced scanning revealed no enhanced signal. The patient, a 56-year-old woman, who was also prone to cerebral infarction, was initially diagnosed with cerebral infarction based on the results of an MRI at a local hospital, which demonstrated the limitation of both plain and enhanced MRI in diagnosing brainstem AA. DWI can reflect the infiltration of tumor tissue into healthy brain tissue via the apparent diffusion coefficient (ADC) value but the value of ADC coexisting in brain tissue is superimposed [8]. DWI is of little value when diagnosing gliomas. Though MRI is the main imaging technique used in assessment of brain tumors, the common MRI findings of brainstem AA are basically the same as those of cerebral infarction, central demyelination and other diseases [9]. Not all gliomas exhibit an enhancement signal [10] and only about 40% of the adult brainstem gliomas show enhancement signal [11]. Magnetic resonance spectroscopy (MRS) is an imaging technology that detects hydrogen atoms in water and fat and thereby the specific energy spectrum of molecules. The accuracy of tumor diagnosis was 85–95% [8]. The brainstem volume is small and closely related to the surrounding adipose and connective tissue. The diagnostic accuracy of MRS in brainstem AA is lower than that in other parts of the brain. The accuracy of MRS can be affected by steroids, mannitol, and other drugs [12]. Initial MRS of this patient did not reveal any differences in Cho/NAA and Cr/NAA values, which led us to consider the diagnosis of demyelinating disease. Four weeks later, the second round of MRI scans revealed that the ratio of Cho/NAA and Cr/NAA increased significantly with the enlargement of the lesion. Two possible reasons are considered for the difference between the two MRS results of the patient: first, the disease progression and early tumor damage to nerve fibers are relatively small with little impact on NAA, and the destruction of nerve fibers increases with disease progression, resulting in a significant reduction in NAA value and an increase in the ratio of Cho/NAA to Cr/NAA. The second is the effect of drugs such as steroids and mannitol on MRS accuracy since steroids can protect the blood–brain barrier and reduce brain edema [13]. Initial MRS of the patient was performed before steroids and mannitol were administered, and the boundary of the focus was unclear, and the edema of the surrounding brain tissue was evident, which could affect the accuracy of MRS measurement. The edema disappeared after treatment and the boundary of the focus was clearly compiled, and the measurement of the MRS spectrum value was more accurate than the earlier test.

^18^F-FDG PET-CT was the first tracer used for of brain tumors, which help in diagnosing tumors and has more advantages than MRI in judging the degree of malignancy of brainstem gliomas [14]. The uptake rate of ^18^F-FDG in gliomas is higher than that in normal brain tissue and the degree of increase is related to the degree of tumor malignancy, which can reach a sensitivity of 80% with almost 100% specificity [15]. When the ratio of ^18^F-FDG to white matter is more than 1.5, or the ratio to gray matter is more than 0.6, the possibility of high-grade gliomas must be considered[8]. PET-CT of this patient revealed that the ^18^F-FDG uptake rate of the focus did not increase, which did not differ significantly from that of the surrounding normal brain tissue, nor did the blood flow increase abnormally in the cerebral blood perfusion map obtained using single-photon emission CT. This is because the metabolism of the brain cadre position is active, and the high variability and heterogeneity of a single tumor to ^18^F-FDG uptake further reduces the ability of PET-CT to detect these lesions, as ^18^F-FDG uptake and blood flow do not differ significantly between the tumor and surrounding brain tissue.

Amino acid PET radiotracers including 3,4-dihydroxy-6-18F-fluoro-l-phenylalanine (^18^F-FDOPA) display superior contrast to 18F-FDG because of low uptake of amino acids in normal brain tissue [16]. A study has shown that both high-grade and low-grade tumors were well visualized with (18)F-FDOPA. The sensitivity for identifying tumors was substantially higher with (18)F-FDOPA PET than with 18F-FDG.

## 4 Conclusion

In the present report, an unusual presentation of AA in the brainstem triggered the misdiagnosis of acute cerebral infarction and demyelinating disease. Although 18F-FDG PET-CT was negative, brain tumor was still considered with atypical imaging characteristics, and finally AA was diagnosed by pathological biopsy. The diagnosis and treatment of this patient reminds us that imaging diagnosis can only be used as an auxiliary diagnosis of the disease, and timely pathological biopsy is very necessary when the clinical manifestations are inconsistent with the image, because ^18^F-FDG PET-CT can be falsely negative even in high-grade glioma.
